# The Evolving Role of Surgical Aortic Valve Replacement in the Era of Transcatheter Valvular Procedures

**DOI:** 10.3390/jcm12165299

**Published:** 2023-08-15

**Authors:** Fernando M. Juarez-Casso, Juan A. Crestanello

**Affiliations:** Department of Cardiovascular Surgery, Mayo Clinic, Rochester, MN 55905, USA; juarezcasso.fernando@mayo.edu

**Keywords:** aortic valve stenosis, surgical aortic valve replacement, transcatheter aortic valve replacement

## Abstract

Surgical aortic valve replacement (SAVR) has long been the standard treatment for severe symptomatic aortic stenosis (AS). However, transcatheter aortic valve replacement (TAVR) has emerged as a minimally invasive alternative; it was initially intended for high-risk patients and has now expanded its use to patients of all risk groups. While TAVR has demonstrated promising outcomes in diverse patient populations, uncertainties persist regarding its long-term durability and potential complications, raising the issue of the ideal lifetime management strategy for patients with AS. Therefore, SAVR continues to play an important role in clinical practice, particularly in younger patients with longer life expectancies, those with complex aortic anatomy who are unsuitable for TAVR, and those requiring concomitant surgical procedures. The choice between TAVR and SAVR warrants personalized decision-making, considering patient characteristics, comorbidities, anatomical considerations, and overall life expectancy. A multidisciplinary approach involving an experienced heart team is crucial in the preoperative evaluation process. In this review, we aimed to explore the current role of surgical management in addressing aortic valve stenosis amidst the expanding utilization of less invasive transcatheter procedures.

## 1. Introduction

**Figure 1:**
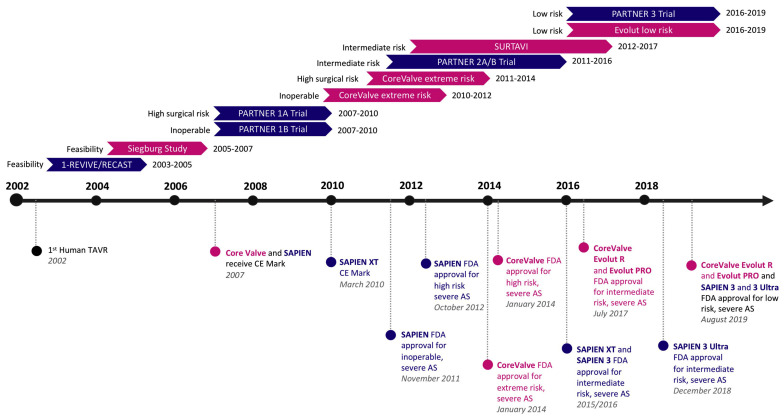
Timeline of milestone trials and FDA approvals for the use of TAVR in patients with severe aortic valve stenosis [15].

Surgical aortic valve replacement (SAVR) has traditionally been considered the standard of care when treating severe symptomatic aortic stenosis (AS) [1,2]. However, transcatheter aortic valve replacement (TAVR) has emerged as a minimally invasive alternative to SAVR [3], revolutionizing the management of patients with severe AS, initially for high-risk patients [4,5,6]. Over the years, TAVR has shown promising clinical outcomes in select patient populations, including those with high and intermediate surgical risk [7,8,9,10,11]. Recently, TAVR has also been approved for use in patients with low surgical risk [12,13,14], expanding its use to patients of all risk groups (Figure 1) [14]. 

Despite the advantages of TAVR, SAVR remains a valuable option in certain clinical scenarios. There is limited evidence on the durability of TAVR beyond 5 years [6,7,16], which suggests that SAVR continues to play a role in treating young symptomatic patients with longer life expectancies (under 65 years of age, according to current guidelines) [14]. In addition, SAVR may be preferred by patients with a complex aortic root anatomy that is unsuitable for TAVR, as well as those who require concomitant surgical procedures.

The choice between TAVR and SAVR is complex decision that should be individualized. A preoperative evaluation should be based on various factors, including patient comorbidities, anatomical considerations, and life expectancy. This process involves shared decision making with the patient and assessment by a multidisciplinary heart team. In this review, we discuss the current role of surgical management in addressing aortic valve stenosis, considering the increasing use of less-invasive transcatheter alternatives. By examining the latest evidence and exploring the evolving landscape, we aim to provide insights into optimal treatment strategies for patients with severe AS.

## 2. Young Low-Risk Patients

As the indications for TAVR expand, the age at which patients are referred for TAVR continues to decrease. The question arises about the best treatment strategy for young low-risk patients with a long-life expectancy. In recent clinical trials, the mean age of low-risk patients undergoing TAVR has ranged from 73 to 79 years [12,13]. Although these trials have provided valuable evidence on the safety of TAVR for low-risk patients over 70 for up to two or three years postoperatively [17,18], the optimal treatment strategy for younger patients remains unclear.

Further, a recent study has provided evidence that the outcomes of SAVR in low-risk patients (STS-PROM < 4%) have consistently improved over time and have even surpassed initial expectations, particularly in high-volume medical centers [19]. These findings raise the possibility of reevaluating existing surgical risk models when making decisions about the optimal timing of intervention and the selection between SAVR and TAVR in young and low-risk patients. Additionally, the Ross procedure has demonstrated promising long-term results over recent years and should be included in the array of surgical options when considering treatment for aortic stenosis in select young patients [20,21,22].

### 2.1. Prosthesis Durability

The assessment of prosthesis durability holds great importance during preoperative evaluation, particularly for young patients with long life expectancies. The long-term functionality of a valve becomes a significant concern as its effectiveness is not indefinite. However, there is a lack of data regarding the long-term durability of TAVR valves. Of note, various factors specific to TAVR, including valve crimping, post-dilation, noncircular implantation, paravalvular leak (PVL), and leaflet thrombosis, can impact its long-term durability [23].

One critical consideration regarding bioprosthetic valves is how their lifespan aligns with the patient′s life expectancy and the possibility of requiring a second valve replacement later in life. Bioprosthetic valve durability is assessed by the rate of bioprosthetic valve deterioration (BVD) and is based on two components: structural valve deterioration (SVD) and non-structural valvular deterioration (non-SVD). SVD is characterized by specific increases in the mean gradient and residual aortic regurgitation (AR), while non-SVD involves severe prosthesis-patient mismatch or PVL [24,25,26]. By comparing both treatment approaches, it may appear that SAVR has a higher rate of valve deterioration, although this conclusion has limitations. Firstly, accurately proving the durability of prosthetic valves is extremely complex since the constant release of new models leads to potentially obsolete data regarding outdated models. Secondly, the diagnostic criteria for SVD are gradient based, which may bias the results against SAVR due to its higher postoperative gradients.

Nonetheless, long-term durability studies of bioprosthetic surgical valves have shown approximately 20% failure rates at 15 years, with younger patients experiencing higher rates of failure [27]. This is why practice guidelines continue to recommend SAVR with mechanical valves for patients under 50 years of age, providing there are no contraindications for anticoagulation [2,28]. These recommendations are also supported by data showing improved survival over a 15-year follow-up period in patients under the age of 55 who received a mechanical aortic valve compared to those who received a bioprosthesis [29]. Avoiding the risk of late reoperations as well as the risk of living with a dysfunctional bioprosthesis, may be responsible for this benefit [30].

As patients who undergo TAVR continue to live longer, our understanding of its long-term durability continues to improve, leading to better-informed decisions regarding the most suitable prosthetic valve for this subgroup of patients. At our current state of knowledge, the optimal choice of valvular prosthesis for young low-risk patients should involve a comprehensive assessment of factors such as the risk of anticoagulation and bleeding, life expectancy, the likelihood of late reintervention due to valve deterioration, and the feasibility of TAVR-in-TAVR and patient preference.

### 2.2. Long-Term Complications

Another important consideration when evaluating the best treatment approach for young and otherwise healthy patients with severe aortic valve stenosis is the risk and quality-of-life implications of long-term complications later after TAVR or SAVR. While both procedures carry risks, TAVR, even with the constant advancements in technology and experience, has a higher risk for certain complications, such as conduction disturbances requiring a pacemaker (PPM) implantation [31,32]. In the PARTNER 3 and Evolut low-risk clinical trials, the risk of 30-day PPM implantation following TAVR ranged from 6.5% to 34%, with lower rates observed in the SAVR group [12,13]. Further, data have shown that PPM implantation following TAVR increases the long-term risk of mortality and rehospitalizations due to congestive heart failure [33,34,35].

Valvular hemodynamics, such as PVL and patient-prosthesis mismatch, are other important long-term considerations for TAVR. Although newer-generation transcatheter heart valves (THV) have reduced rates of moderate and severe PVL following TAVR, more than one-third of patients still experience mild regurgitation postoperatively [36]. Even mild PVL can have a cumulative effect over time and may be associated with an increased risk of death late after TAVR [36]. On the other hand, severe patient-prosthesis mismatch is more common after SAVR and is associated with a higher risk of all-cause mortality and rehospitalization at 5 years [37].

In high-risk patients, TAVR carries a higher risk of stroke at 30 days and 1 year compared to SAVR [4,5]. However, this risk is less clear for intermediate-risk and low-risk patients, with variations observed depending on the type of valve used [8,9,12,13]. A recent meta-analysis found a trend against TAVR in terms of stroke risk, although this difference was not statistically significant. Despite this, it remains crucial to discuss the risk of stroke with patients, particularly younger individuals, as it can significantly affect their quality of life and increase the chances of operative complications and long-term mortality.

While the risk of these complications should not be the sole factor guiding the treatment approach for low-risk patients with AS, it is important to discuss the potential long-term implications of TAVR with patients. Hence, it is reasonable for younger low-risk patients to choose SAVR and avoid some of these potential complications.

### 2.3. Lifetime Management

**Figure 2:**
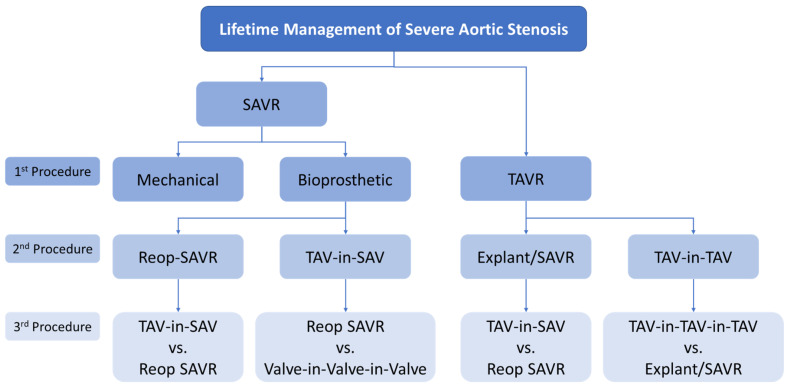
Procedure sequence in the lifetime management of patients with severe aortic valve stenosis.

Another important factor when evaluating TAVR in young patients is to consider the possibility of future procedures, such as repeat valve replacement or coronary access for diagnostic or interventional procedures. This concept of “lifetime management” should be considered when determining the optimal treatment approach for the initial procedure. The expanding use of valve-in-valve (VIV) procedures has provided additional options for second and third valve replacement procedures. It is our opinion that the highest-risk procedure should be performed first while the patients are still young to minimize the need for surgical interventions in older and higher-risk patients in the future [38]. Careful evaluation and planning on the order and sequence of treatment options is essential (Figure 2) [39,40,41,42]. 

Recent data on reoperations and the valve explanation following TAVR highlight the significant surgical risks involved [43,44]. These reoperations are more common in intermediate and low-risk patients and are associated with acceptable rates of postoperative complications (when excluding emergency cases) [43]. Notable, valve explanations have posed a greater challenge in patients with a native TAVR valve compared to those who underwent TAVR-in-SAVR, with a substantial proportion (13% to 23%) of patients requiring aortic root replacement [43,44]. Reoperations in patients with a native TAVR valve have higher rates of operative and mid-term mortality; however, a lower cumulative incidence of post-implant valve reintervention has been found compared to patients with a TAVR-in-SAVR procedure [43]. It is also worth mentioning that many patients undergoing reoperation after TAVR require surgery for indications other than aortic valve disease or necessitate additional procedures in addition to AVR. 

These findings highlight the importance of careful patient selection and the appropriate consideration of the valve type used in the initial aortic valve procedure, especially in younger and lower-risk patients. For subsequent procedures following initial aortic valve replacement, TAVR-in-SAVR is usually preferred for older, higher-risk patients or with multiple previous sternotomies. TAVR-in-TAVR represents a valid alternative for high-risk patients, although further evidence is required to assess the durability and hemodynamic performances of the second TAVR valve [45]. On the other hand, redo-SAVR is a better alternative for younger low-risk patients, those with small prostheses or a high risk of coronary obstruction, and cases of infectious endocarditis.

The expanding use of TAVR in younger low-risk patients also brings attention to coronary artery access for diagnostic or interventional procedures, particularly with self-expanding valves. Challenges to accessing the coronary arteries can be related to the type and position of the valve (implantation height and alignment of the TAVR and native commissures), the anatomy of the sinus of Valsalva, sino-tubular junction, and the height of the coronary artery ostia. The cumulative incidence of percutaneous coronary artery intervention (PCI) after TAVR over a ten-year period is 0.6% for patients without coronary artery disease (CAD), 3.4% for those with single-vessel disease, and 7.4% for individuals with multivessel disease [46]. Importantly, patients experiencing ST-segment elevation myocardial infarction after TAVR face longer door-to-balloon times, increased PCI failure rates, and higher mortality compared to non-TAVR patients [47]. The realignment of TAVR commissures with native ones can facilitate future coronary access, although TAVR-in-TAVR procedures also introduce additional complexity due to the height of the new valve and the alignment of the stent frames [48].

These findings underscore the importance of careful clinical judgment and candidate selection in TAVR procedures. The evaluating team should be mindful of the concept of “lifetime management” when selecting the most suitable valve for the initial aortic valve procedure, particularly in younger and lower-risk patients.

## 3. Anatomical Challenges

SAVR remains a safe and reliable option for patients with challenging anatomical features that make TAVR more challenging, such as bicuspid aortic valve, heavy annular-LVOT calcification, and low coronary heights. Additionally, SAVR can also be considered for certain extended indications, including early treatment in patients with moderate or asymptomatic stenosis, as well as pure AR, small aortic annulus root, and a lack of transfemoral access. These factors highlight the versatility and effectiveness of SAVR when addressing complex anatomical situations where TAVR might not be the optimal choice.

### 3.1. Bicuspid Aortic Valve

Patients with a bicuspid aortic valve (BAV) typically belong to a younger and lower-risk population with an extended life expectancy. The unique characteristics of BAV, such as excessive raphe and leaflet calcification, as well as the presence of ascending aortic aneurysms, pose significant challenges in TAVR procedures. Studies comparing TAVR and SAVR in BAV patients are limited; however, some of the available evidence suggests comparable outcomes in carefully selected patients [49,50]. However, a recent meta-analysis reported higher rates of conversion to surgery, PVL, and patient-prosthesis mismatch in BAV patients treated with TAVR [51]. Further research is needed to guide the selection of the THV type and size for diverse BAV anatomies.

While TAVR has shown favorable early results in low-risk patients with bicuspid aortic valve stenosis [49], longer-term outcomes and randomized clinical trials comparing TAVR with surgery are necessary before considering changes in their clinical guidelines. It is worth noting that approximately 2% of the general population has a bicuspid aortic valve, and up to 49% of patients undergoing SAVR for AS have bicuspid anatomy [52]. Anatomical considerations in bicuspid valves, such as raphe location and length, the degree of symmetry/asymmetry of calcification, the size of the sinuses of Valsalva, and the presence of left ventricular outflow tract calcium, further complicate the choice between TAVR and SAVR [53,54]. In this patient population, the risk of encountering periprocedural complications is significantly higher. These complications include annular rupture, asymmetric valve deployment, PVL, conversion to open surgery, and operative mortality [55,56].

In the realm of TAVR for patients with BAV, careful patient selection, thorough pre-procedural planning, and specific intra-procedural techniques are vital to ensure optimal device implantation and minimize complications. It is important to note that for patients with BAV who are younger (aged <70 years) or have aortic root dilation (≥45 mm and aged <75 years), surgery should still be considered the preferred treatment option [57]. To expand our understanding of TAVR in BAV patients, randomized trials comparing TAVR and SAVR in older patients at low and intermediate surgical risk are warranted. 

### 3.2. Annular and LVOT Calcification 

The assessment of annular and left ventricular outflow tract (LVOT) calcification severity lacks objective or universally accepted criteria. In both the PARTNER 3 and Evolut Low-Risk trials, the final decision to include patients in this study was determined by a committee that analyzed clinical and imaging data; in the PARTNER 3 trial, 38% of patients who did not pass the review were deemed ineligible due to severe LVOT calcium, while 8% of patients were disqualified because of severe LVOT calcium in the Evolut Low-Risk trial [12,13].

The presence of severe calcification has been consistently associated with worse outcomes after TAVR, including risks of annular rupture, PVL, and stroke [58,59,60,61]. The risk of annular rupture significantly increases with the use of oversized balloon-expanded valves and self-expanding valves that require post-dilation. Annular rupture is a catastrophic complication that is associated with a remarkably high risk of mortality, with some publications reporting mortality rates exceeding 50% [58,61].

Heavy annular and LVOT calcification has also been associated with increased rates of moderate or severe PVL following TAVR. Early TAVR trials reported higher incidences of moderate or more severe PVL, which were likely influenced by factors such as heavy annular and LVOT calcification, less advanced THV technology, and the lower utilization of post-dilation [62,63]. In recent low-risk trials, the incidence of moderate or more severe PVL has ranged from 0.6% to 3.6%, reflecting improvements in patient selection, THV advancements, and the increased use of post-dilation [12,13]. The Nordic Aortic Valve Intervention (NOTION) study, which did not systematically exclude patients with heavy annular and LVOT calcification, reported a higher rate of moderate or more severe PVL at approximately 15% in the low-risk subset [64].

Additionally, calcium and atheromatous embolization play a significant role in the occurrence of strokes after TAVR. Various techniques performed during TAVR, such as manipulating wires and catheters in the aortic arch, pre-TAVR balloon aortic valvuloplasty, positioning the THV, and post-dilation, can dislodge calcific deposits, leading to embolic events. The effectiveness of cerebral embolic protection devices in reducing the risk of stroke during TAVR remains a topic of debate due to conflicting data [65,66,67]. Initially, a stroke was a major concern associated with TAVR; however, in recent low-risk trials, the rates of disabling strokes within 30 days has significantly decreased from 6% to less than 2% [12,13]. It is worth noting that post-dilation, which is performed in approximately 20% to 30% of patients in low-risk trials [12,13], has been linked to a 2.5-fold increase in the risk of stroke [68,69]. This highlights the importance of carefully considering the potential risks and benefits of post-dilation in TAVR procedures.

To ensure optimal outcomes, it is crucial to carefully consider alternative treatment options, such as SAVR, for patients presenting with heavy and asymmetric calcification patterns. This is particularly important as such patterns have been associated with increased risks of annular rupture, significant PVL, and stroke. SAVR offers advantages in addressing heavy calcification directly during the procedure. By visualizing and surgically addressing the calcification, SAVR significantly reduces the incidence of significant PVL and improves postoperative outcomes. Thus, in cases involving patients with extensive and asymmetric calcification, SAVR should be strongly considered as a preferred treatment option.

### 3.3. Low Coronary Height

Although coronary obstruction is a rare occurrence in initial TAVR procedures, this serious complication carries a high risk of procedural morbidity and mortality [70]. Obstructions typically arise when the valve leaflets and associated calcium are displaced, leading to the blockage of the coronary arteries. Obstruction can also arise from sinus sequestration, particularly when the sinotubular junction is low and has a small diameter. In such instances, the displaced native or transcatheter (in valve-in-valve procedures) valve leaflets can impede the flow into the sinus of the Valsalva. Advancements in identifying patients at risk of coronary obstruction have significantly improved; those with coronary ostia positioned less than 10 mm above the annulus, effaced sinuses, and a narrow and/or low sinotubular junction face a higher risk of occlusion [70]. The left coronary ostium is the most frequently affected, especially in women, patients with a small aortic annulus and effaced sinuses, those with BAV, and following TAVR-in-SAVR procedures.

Coronary obstruction is more common in TAVR-in-SAVR, with a 2–3 times higher incidence compared to the initial procedure [71]. Further, in TAVR-in-TAVR cases, the failed THV can create a “tube graft” where the leaflet of the initial THV becomes trapped between two THV frames, obstructing coronary access and flow [48]. The extent of entrapment depends on the frame height and position of the second THV, impacting valve performance, durability, and coronary artery access.

The use of a chimney stent, which acts as a protective measure for at-risk coronary arteries following TAVR, has demonstrated favorable mid-term survival rates [72]. However, long-term data and concerns relating to coronary re-accessibility persist in these patients. The bioprosthetic aortic scallop intentional laceration (BASILICA) technique, involving a deliberate incision of the aortic scallop to prevent iatrogenic coronary artery obstruction, has shown promise in preventing such obstructions. Nevertheless, these studies primarily focused on intermediate and high-surgical-risk patients [73]. If this complication occurs, prophylactic stenting in the potentially affected coronary arteries is an available treatment option, although it carries a higher risk of stent compression and thrombosis [74,75].

SAVR should continue to be the preferred treatment approach for low-surgical-risk patients with severe AS who are at significant risk of TAVR-induced coronary obstruction.

### 3.4. Isolated Aortic Regurgitation

In patients with severe symptomatic AR, SAVR remains the standard of treatment since conservative management has proven to be ineffective in these cases. However, some patients face high surgical risks and postoperative mortality, limiting their therapeutic options. Patients with isolated AR often have less leaflet calcification and significant dilation of the ascending aorta and valvular annulus, making valve anchoring and positioning challenging during TAVR. These anatomical factors result in a higher incidence of moderate-to-severe PVL following TAVR [76]. A recent publication reported a device implantation success rate of 72%, an early survival rate of 66%, and a clinical effectiveness rate of 61% after TAVR in patients with severe isolated AR [77]. The success rate of device implantation and clinical efficiency was higher with newer models than those of the first-generation device, and the incidence of moderate/severe PVL was also significantly decreased. 

Despite these advancements, TAVR for AR remains off-label in the United States, as the etiology of regurgitation differs from AS, and associated anatomical factors may pose significant challenges. SAVR is often a more suitable therapy for low-surgical-risk patients with AR due to insufficient calcium for valve anchoring, a potentially dilated aortic root, and the risk of PVL. Registry data indicate that even with newer-generation prostheses, there are still risks, such as open conversion, the need for a second THV, pacemaker implantation, and moderate or greater residual regurgitation [78]. Until more data are available, SAVR remains the treatment of choice for low-surgical-risk patients with isolated AR [77,78].

### 3.5. Small Aortic Annulus

There is a lack of universal consensus regarding the optimal cut point to define a small aortic annulus. Some authors propose an annular diameter of 21–23 mm or an annular area of <430 m^2^ [79,80]. Approximately 17% of patients with aortic stenosis present with a smaller aortic annulus, primarily in older women [81]. This condition has significantly increased the risk of patient-prosthesis mismatch, which is, in turn, associated with worse long-term outcomes and higher mortality rates [82,83,84]. While TAVR allows for oversizing the THV, a reduction in the risk of severe mismatch compared to SAVR, patients with small aortic annulus receiving TAVR still experience a 4–20% rate of severe patient-prosthesis mismatch [85,86]. Oversizing THV in these patients can also increase the risk of annular rupture and coronary obstruction [58,70].

SAVR in low-risk patients offers the option of aortic root enlargement, which significantly reduces the incidence of post-operative patient-prosthesis mismatch by up to 50% and improves short- and long-term outcomes [87]. Enlarging the aortic root during SAVR in younger patients allows for the use of a larger valve during future TAVR-in-SAVR in cases where the first bioprosthesis fails. In younger low-risk patients with a smaller aortic annulus who are at low risk of bleeding, SAVR with a mechanical prosthesis can also be considered to obviate the need for a second valve procedure and further limit the risk of patient-prosthesis mismatch. Although ongoing randomized studies like the SMART Trial (NCT 04722250) and the VIVA Trial (NCT 03383445) are comparing TAVR and SAVR in small aortic annulus patients, SAVR is expected to maintain a relevant role in low-surgical-risk patients with severe AS and a small aortic annulus.

## 4. Need for Concomitant Procedures

The Evolut Low-Risk and PARTNER 3 low-risk trials revealed a notable presence of coexisting coronary artery disease (CAD) among low-risk patients undergoing TAVR, with 13% and 28% having a history of prior percutaneous coronary intervention (PCI) [12,13]. Of note, patients with complex CAD or significant left main disease were excluded from both trials. Previous studies have shown that patients with comorbid CAD have worse outcomes after TAVR compared to those without CAD [88,89]. To improve outcomes in patients with multivessel CAD, current guidelines recommend concomitant coronary artery bypass grafting (CABG) during SAVR [28]. However, there is no consensus as to the specific lesions that should be revascularized before TAVR, and ongoing prospective trials aim to address this uncertainty [90].

In addition to CAD, concurrent valvular diseases influence the choice between TAVR and SAVR in low-risk AS patients. Current guidelines recommend mitral valve surgery for patients with asymptomatic moderate to severe primary mitral regurgitation when undergoing cardiac surgery for other indications [28]. Similarly, the presence of a dilated ascending aorta, particularly in low-risk patients with bicuspid AS, favors SAVR with aortic root repair (however, managing patients with borderline or mildly dilated aortas where interval imaging is not available remains unclear). Additionally, patients with chronic atrial fibrillation may also benefit from surgery by undergoing concomitant atrial fibrillation ablation with a MAZE procedure. Recent data have shown that patients with severe AS and persistent AF who undergo SAVR or TAVR without AF ablation have a higher risk of long-term mortality [91].

The presence of significant mitral and/or tricuspid valve disease is common in patients undergoing TAVR and is associated with a considerably worse prognosis if left untreated [92,93,94,95]. In general, the current guidelines recommend SAVR as the preferred treatment strategy for patients with bicuspid aortic valve (BAV) and with aortopathy greater than 4.5 cm, those with multivessel disease or left main coronary artery involvement, patients with severe mitral and/or tricuspid valve disease, and patients with infected endocarditis requiring the surgical removal of infected tissue [2,28].

## 5. Conclusions

**Figure 3:**
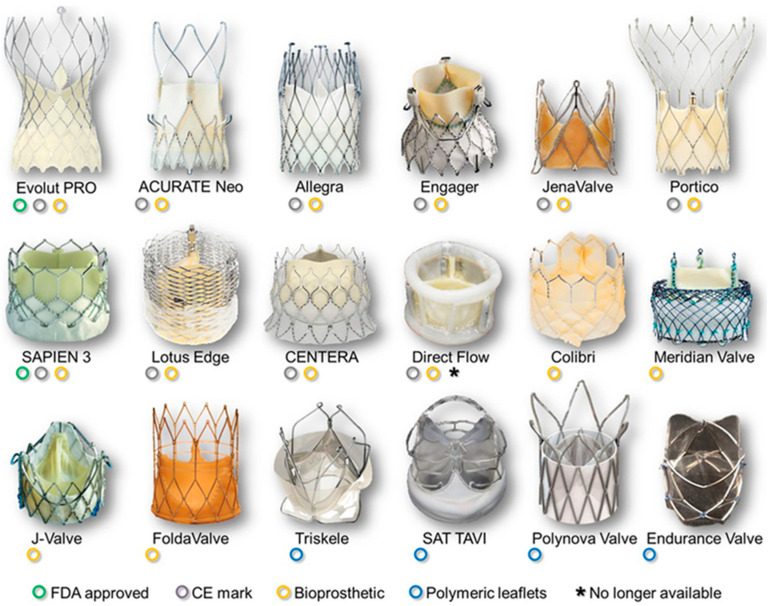
Commercially available and under-investigation TAVR models [96].

TAVR has been a groundbreaking development in cardiovascular medicine, currently representing the predominant treatment modality for patients with severe AS. TAVR offers a safe and reproducible procedure with low risk, rapid recovery, and promising long-term outcomes. It has demonstrated efficacy across various surgical risk categories, benefiting the vast majority of patients. As the utilization of TAVR continues to grow, there is great potential for ongoing improvement driven by increasing operative experience, technological advancements, and a growing body of quality evidence (Figure 3).

Although significant efforts have been made to address the primary challenges associated with TAVR, there are still lingering questions that require further investigation and attention. It is essential for healthcare providers to acknowledge the complementary nature of TAVR and SAVR and the importance of a multidisciplinary “Heart Team” in the patient selection process. Factors such as age, life expectancy, surgical risk, concurrent cardiac conditions, aortic root anatomy, long-term durability, procedural sequencing (lifetime management), and coronary access should be carefully evaluated to ensure optimal outcomes for all patients.

## Data Availability

Data sharing not applicable.
